# Progranulin Oncogenic Network in Solid Tumors

**DOI:** 10.3390/cancers15061706

**Published:** 2023-03-10

**Authors:** Elisa Ventura, Giacomo Ducci, Reyes Benot Dominguez, Valentina Ruggiero, Antonino Belfiore, Elena Sacco, Marco Vanoni, Renato V. Iozzo, Antonio Giordano, Andrea Morrione

**Affiliations:** 1 Sbarro Institute for Cancer Research and Molecular Medicine, Center for Biotechnology, Department of Biology, College of Science and Technology, Temple University, Philadelphia, PA 19122, USA; 2 Department of Biotechnology and Biosciences, University of Milano-Bicocca, 20126 Milan, Italy; 3 SYSBIO (Centre of Systems Biology), ISBE (Infrastructure Systems Biology Europe), 20126 Milan, Italy; 4 Department of Pharmacological Sciences, Master Program in Pharmaceutical Biotechnologies, University of Padua, 35131 Padua, Italy; 5 Department of Clinical and Experimental Medicine, Endocrinology Unit, University of Catania, Garibaldi-Nesima Hospital, 95122 Catania, Italy; 6 Department of Pathology, Anatomy and Cell Biology, Translational Cellular Oncology Program, Sidney Kimmel Cancer Center, Sidney Kimmel Medical College at Thomas Jefferson University, Philadelphia, PA 19107, USA; 7 Department of Medical Biotechnologies, University of Siena, 53100 Siena, Italy

**Keywords:** progranulin, solid tumors, RTKs

## Abstract

**Simple Summary:**

The growth factor progranulin plays an important pro-tumorigenic role in several solid tumors and a growing number of studies suggest diagnostic and prognostic values for progranulin in many tumor types. Progranulin exerts its pro-tumorigenic action by affecting both tumor cells and the tumor microenvironment. However, the details of progranulin pro-oncogenic function are not fully elucidated and recent evidence suggests a strong context-dependency of progranulin signaling. In this review, we will summarize the current evidence supporting the progranulin pro-oncogenic role, with a particular focus on what is currently known about progranulin molecular mechanisms of action in cancer.

**Abstract:**

Progranulin is a pleiotropic growth factor with important physiological roles in embryogenesis and maintenance of adult tissue homeostasis. While-progranulin deficiency is associated with a broad range of pathological conditions affecting the brain, such as frontotemporal dementia and neuronal ceroid lipofuscinosis, progranulin upregulation characterizes many tumors, including brain tumors, multiple myeloma, leiomyosarcoma, mesothelioma and epithelial cancers such as ovarian, liver, breast, bladder, adrenal, prostate and kidney carcinomas. The increase of progranulin levels in tumors might have diagnostic and prognostic significance. In cancer, progranulin has a pro-tumorigenic role by promoting cancer cell proliferation, migration, invasiveness, anchorage-independent growth and resistance to chemotherapy. In addition, progranulin regulates the tumor microenvironment, affects the function of cancer-associated fibroblasts, and modulates tumor immune surveillance. However, the molecular mechanisms of progranulin oncogenic function are not fully elucidated. In bladder cancer, progranulin action relies on the activation of its functional signaling receptor EphA2. Notably, more recent data suggest that progranulin can also modulate a functional crosstalk between multiple receptor-tyrosine kinases, demonstrating a more complex and context-dependent role of progranulin in cancer. Here, we will review what is currently known about the function of progranulin in tumors, with a focus on its molecular mechanisms of action and regulation.

## 1. Introduction

Progranulin is a pluripotent growth factor with important roles in several physiological processes. Progranulin is expressed in both the embryo and placenta, where it modulates embryo growth [1] and implantation [2], as well as placenta formation [3]. In adult tissues, progranulin regulates tissue regeneration [4,5], promotes angiogenesis [6], modulates the immune response [7,8] and is implicated in host defense against bacterial infections [8,9]. In addition, progranulin is a key neurotrophic factor as, in fact, it promotes neuronal survival and neurite growth [10,11], modulates neuroinflammation [12] and regulates lysosome function in neurons [13,14]. On the other hand, progranulin dysregulation is involved in several diseases [15] and therefore has attracted attention as a potential therapeutic target [16]. Progranulin mutations and heterozygous or homozygous loss are associated with various and severe pathologies affecting the brain, including frontotemporal dementia and lysosomal storage diseases [17,18,19]. Dysregulated progranulin is also implicated in autoimmune diseases [20]. Progranulin is overexpressed in several cancer types, including hematological malignancies, where it exerts a critical role in tumor progression. In this review, we focus on the role of progranulin in solid tumors, with a particular attention to the known receptors and signaling pathways that are implicated in progranulin pro-oncogenic action.

## 2. Progranulin Structure and Nomenclature

**Figure 1:**
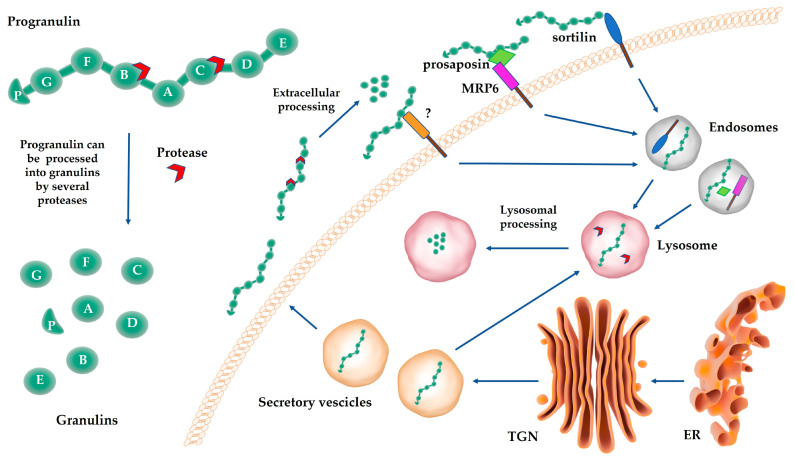
Progranulin structure, processing and trafficking. The growth factor progranulin is a modular protein containing seven and half non-identical, cysteine-rich tandem repeats, known as granulin domains. Progranulin can be processed by several proteases into single granulin modules. Progranulin is released into the extracellular environment by regulated exocytosis. Extracellular progranulin can be internalized in a sortilin- or prosaposin-dependent manner and sorted into lysosomes but can also reach the lysosomes diverting from the secretory pathway. In lysosomes, progranulin is processed by cathepsin L into granulins. Whether progranulin might be endocytosed in a sortilin- and prosaposin-independent manner through the binding to other receptors is still not fully defined. ER: endoplasmic reticulum. TGN: trans-Golgi network.

The growth factor progranulin is a modular protein containing seven and half non-identical, cysteine-rich tandem repeats, known as granulin domains. Granulins A-G are full modules, while p or paragranulin is the N-terminal half-module (Figure 1). The granulin domain is evolutionary highly conserved [21] and has a unique structure consisting of four β-hairpins held together by six disulfide bridges [22,23]. Progranulin homologs can be found in a broad range of living organisms, ranging from plants to mammals [21]. In invertebrates and fish, progranulin is coded by multiple *GRN* genes, whereas in the majority of tetrapodes and in all mammals, progranulin is coded by a single gene. In humans, the *GRN* gene is located on chromosome 17 (17q12.31) and contains a 5′ non-coding exon and 12 coding exons. Each granulin repeat is coded by two adjacent exons [21].

Progranulin is secreted by regulated exocytosis (Figure 1) as a highly glycosylated protein of around 70–80 kDa [24], as soluble protein or in exosomes [25]. Progranulin N-glycosylation can occur on five different N-glycosylation sites with a prevalent addition of fucosylated oligosaccharides [26]. Secreted progranulin can be processed into single granulin modules of around 6 kDa (Figure 1), known as granulins, by various extracellular proteases, including matrix metalloproteases (MMP) MMP-9, MMP-12 and MMP-14 [27], elastase [28,29], proteinase 3 [29] and ADAM metallopeptidase with thrombospondin type 1 motif 7 (ADAMTS7) and 12 (ADAMTS12) [30]. On the other hand, progranulin binding to the high-density lipoprotein (HDL)/apolipoprotein A-I complex [31] or the secretory leukocyte protease inhibitor (SLPI) [28] protects progranulin from proteolytic cleavage, thereby preserving progranulin precursor activity [28].

Granulins are biologically active but often exert opposing functions when compared to the full-length progranulin precursor [22,32], and the levels of extracellular proteases and protease inhibitors determine the relative abundance of progranulin and granulins in the extracellular environment. In addition, there are progranulin fragments with an intermediate size between progranulin and granulins, which are active as well, such as the epithelial transforming growth factor (TGFe) [33].

Extracellular progranulin is internalized by endocytosis and sorted into lysosomes (Figure 1). Interestingly, progranulin can also reach the lysosomes diverting from the secretory pathway [13,34] (Figure 1). In lysosomes, progranulin is processed by cathepsin L into granulins, which are quite stable in this subcellular compartment [35,36]. However, the potential lysosomal function of granulins is still elusive [35,36].

Since granulins and progranulins were initially discovered by different groups in different contexts, the original nomenclature was quite confusing. Granulins were originally identified as components of rat granulocytes granules and therefore called granulins [37]. Simultaneously, they were identified in rat kidneys and called epithelins [38]. Genetic studies later revealed that granulins and epithelins were coded by a single gene and named either progranulin, proepithelin or granulin-epithelin precursor (GEP) [39,40]. Guinea pig progranulin was first isolated from the acrosome and called acrogranin [41]. Progranulin was also identified as a secreted growth factor from murine adipocytic teratoma PC cells and named PC-cell-derived growth factor (PCDGF) [42,43,44,45,46], also known as glycoprotein 88 kDa (GP88). Further studies demonstrated that all these proteins were coded by the same gene [42,47].

## 3. Progranulin Binding Proteins

Progranulin pleiotropic action depends on its modular structure and its ability to interact with a broad range of molecules, including extracellular soluble proteins, components of the extracellular matrix, membrane proteins and proteins of the endoplasmic reticulum (ER)/Golgi/lysosome network. The list of proteins interacting with progranulin is continuously growing. Recently, new progranulin-binding proteins have been identified using the ligand receptor capture technique in the neuron-like cell line NCS-34, but the biological relevance of these novel interactions is still unknown [48]. Progranulin-binding proteins can be divided into three main categories: (1) extracellular proteins; (2) membrane proteins; and (3) ER/Golgi/lysosome network proteins. In addition, it has been reported that progranulin and some granulin repeats can localize to the nucleus, where they interact with the Tat/positive transcription elongation factor b (P-TEFb) and inhibit Tat transactivation [49,50].

### 3.1. Progranulin Interaction with Extracellular Proteins

Secreted progranulin not only interacts with various extracellular proteases, which are responsible for progranulin processing into granulins, as well as with proteins protecting progranulin from proteolytic degradation, but also with different components of the extracellular matrix (ECM), including perlecan [51,52], cartilage oligomeric matrix protein (COMP) [53] and extracellular matrix protein 1 [54]. The interaction of progranulin with perlecan is mediated by granulin modules F and B and the first two-laminin- and epidermal growth factor-like repeats of progranulin and perlecan, respectively [51], and modulates tumor angiogenesis [51]. Progranulin interaction with COMP, mediated by the granulin module A, potentiates progranulin-dependent stimulation of chondrocyte proliferation [53], while the association of progranulin with extracellular matrix protein 1 negatively regulates chondrogenesis and endochondral ossification [54].

### 3.2. Progranulin Interaction with Membrane Proteins and Membrane Receptors

Progranulin can bind several membrane proteins and cell membrane receptors, such as sortilin [13], prosaposin [55], tumor-necrosis factor receptor (TNFR) 1 and 2 [7], DR3 [56], four Notch receptors [57], DLK1 [58], EphA2 [59], RET [48] and Toll-like receptor (TLR)9 [9], and these interactions are highly context-dependent.

Sortilin and prosaposin are principally responsible for progranulin lysosomal trafficking. Sortilin belongs to the vacuolar protein sorting 10 (Vps10) family of receptors and its binding to progranulin leads to progranulin endocytosis and trafficking into lysosomes [13] (Figure 1). Secreted progranulin can interact with soluble prosaposin, in turn mediating progranulin internalization and lysosomal sorting by interacting with the mannose-6-phosphate receptor (MRP6) or the low-density lipoprotein receptor-related protein 1 (LRP1) [55] (Figure 1). Both sortilin and prosaposin can mediate progranulin delivery into lysosomes from either the extracellular space or the secretory pathway [13,34]. Evidence suggests that the interactions of progranulin with sortilin and/or prosaposin are particularly relevant in neurological cells [60]. Whether the interaction of progranulin with other membrane receptors, including RTKs, leads to progranulin internalization is not well established (Figure 1).

Progranulin binds to TNFR1 and TNFR2 on immune cells, mostly macrophages and Tregs, competing with TNF-alpha for receptor binding, thereby inhibiting TNF-alpha pro-inflammatory activity [7]. It is important to mention to that progranulin interaction with TNFRs remains controversial, since other groups failed to confirm a direct binding of progranulin to TNFRs [61,62,63]. These discrepancies might be due to technical differences in the surface plasmon resonance (SPR) experimental approaches used by different groups [64]. In addition, progranulin binds to the TNFR1 homolog death receptor 3 (DR3), thereby inhibiting DR3 binding to its natural ligand TNF-like ligand 1 (TL1A) [56].

Progranulin binds to Notch receptors by interacting with the extracellular domain of the receptor, as demonstrated for the interaction with Notch1 [57]. Progranulin activates Notch signaling pathways, promoting peripheral nerve regeneration and motor function recovery [57]. In addition, progranulin interacts with DLK1, a modulator of the Notch signaling pathway, but the biological relevance of this interaction is unknown [58].

In bladder cancer cells, progranulin binds to and activates ephrinA1-independent EphA2 non-canonical signaling [59] favoring tumor progression, while in the neuron-like cell line NSC-34, progranulin binds to RET and promotes its tyrosine-phosphorylation [48].

Finally, progranulin binds to both TLR9 and CpG oligonucleotides (CpG-ODNs) in immune cells and endosomes, favoring TLR9 and CpG-ODNs interaction and potentiating the innate immune response to bacterial infections [9]. Notably, it has been reported that progranulin can activate other receptor-tyrosine kinases, including members of the Eph family, such as EphA4 and EphB2 [48,59], EGFR [48,59,65], ErbB2 [48] and RYK [65]. However, it is not known whether progranulin activates these receptors by direct binding or indirectly by activating functional cross-talks.

The domains responsible for progranulin interaction with some of its membrane binding partners have been characterized [16] and referenced herein. Progranulin interaction with TNFR1, TNFR2 and DR3 is mediated by the granulin modules A, C and F and the linkers P3, P4 and P5, while domains A, C, D and E allow the interaction with TLR9 and CpG-ODNs [16]. Progranulin binds to sortilin through the last three amino acids in its C-terminal (QLL) [66]. Multiple granulin domains, mostly granulins D and E, bind to the linker region connecting saposins B and C in the prosaposin molecule [67]. On the receptors side, the domains involved in progranulin binding are known only for TNF receptors and DR3 [68]. Indeed, it has been demonstrated that progranulin binds the cysteine-rich domains (CRD)2 and 3 of TNF receptors [68]. Considering that both CRD and EGF-like domains can bind to progranulin and that at least one of these domains is part of the extracellular region of all known progranulin-binding receptors, it is possible that CRD and EGF-like domains are more likely involved in progranulin interactions with other receptors than TNFR.

### 3.3. Progranulin Binding Partners Belonging to the ER/Golgi/Lysosome Network

Intracellular progranulin mostly localizes in the endoplasmic reticulum and lysosomes [69]. In the ER, progranulin binding partners include several chaperones, such as endoplasmic reticulum protein (ERp)5, ERp57 and ERp72, heat-shock protein 70 (HSP70), GRP94, binding immunoglobulin protein (BiP), calreticulin and protein disulfide isomerase (PDI) [69] and references therein. It is believed that these chaperones assist in progranulin folding and secretion [69]. In lysosomes, progranulin acts as a co-chaperone by interacting with various hydrolases, such as glucocerebrosidase (GCase), cathepsin D (CSTD) and β-hexosaminidase (HexA) [69]. The relevance of progranulin function as a lysosomal protein is exemplified by the phenotypes associated with progranulin loss, as reviewed by Chitramuthu et al. [17]. Indeed, progranulin deficiency is usually associated with lysosomal disfunctions with progranulin homozygous loss causing cerebroid lipofuscinosis, a severe lysosomal disorder [17]. On the contrary, *GRN* haploinsufficiency leads to frontotemporal dementia (FTD), a disorder characterized by the neurodegeneration of the frontal and temporal lobes, and lysosome disfunction associated with the presence of neuronal inclusions containing fragments of ubiquitinated TDP-43 [17].

## 4. Progranulin in Solid Tumors

Progranulin was originally identified as a soluble factor promoting cancer progression and regulating wound healing [4,70,71,72]. Later studies demonstrated that progranulin is upregulated in many solid tumors, where it promotes tumor cell proliferation, migration, invasion, adhesion, in vivo tumor formation and maintenance of cancer stem cells (CSC) (Table 1). In addition, progranulin contributes to the establishment and maintenance of a tumor microenvironment (TME) that favors tumor progression by modulating the function of several cellular components of the TME, including endothelial cells, immune cells and cancer-associated fibroblast (CAF) (Table 1) [73].

### 4.1. Progranulin Autocrine Function on Tumor Cells

#### 4.1.1. Progranulin and Tumor Cell Proliferation, Migration and Invasion

The role of progranulin in promoting tumor cell proliferation and motility has been well established. Progranulin promotes cell proliferation in many tumor models, as extensively reviewed by Bateman et al. and Arechavelata-Velasco et al. [15,74], but the molecular mechanisms are not completely understood. Some evidence suggests that progranulin can modulate CDK4 activity, cyclin D1 and cyclin B levels, as well as c-myc function by activating the AKT and MAPK signaling pathways [74,75,76]. In addition, recently published data support the evidence of crosstalk between progranulin and the TGF-β signaling pathway, which affects cell proliferation [77].

A critical role for progranulin in mediating cell motility has been demonstrated in many tumor models with multiple mechanisms proposed. Indeed, progranulin promotes an epithelial-to-mesenchymal transition (EMT) process, thereby favoring the acquisition of a highly migratory and invasive phenotype [15,74].

**Figure 2:**
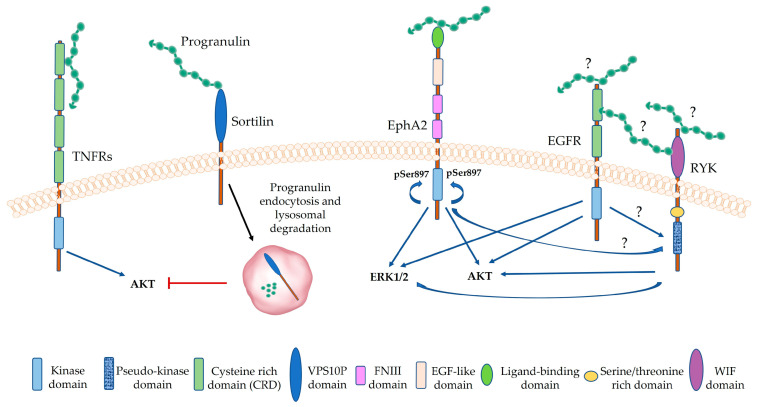
Progranulin signaling in cancer. Progranulin oncogenic signaling is highly dependent on progranulin-dependent activation of AKT and/or MAPK signaling pathways. In colorectal cancer, progranulin promotes AKT activation in a TNFR2-dependent manner. In prostate cancer, sortilin acts as a negative regulator of progranulin by promoting progranulin internalization and degradation, leading to the inhibition of the AKT pathway. In turn, progranulin mediates sortilin ubiquitination and degradation to sustain its pro-oncogenic activity. In bladder cancer, progranulin binds to and activates EphA2, leading to AKT and MAPK activation. In turn, AKT and MAPK sustain EphA2 phosphorylation at Ser897. In mesothelioma, progranulin-dependent activation of the AKT and MAPK signaling pathways relies on EGFR and RYK. Progranulin directly interacts with TNFRs, sortilin and EphA2. Whether progranulin promotes EGFR and RYK phosphorylation and activation directly by physically interacting with the receptors, or in an indirect manner, or whether progranulin promotes the formation of a complex including EGFR, RYK and EphA2 requires further investigation.

In bladder cancer, progranulin promotes cell migration and invasion by inducing the formation of a molecular complex containing focal adhesion kinase (FAK) and paxillin, in an ERK1/2-dependent manner [78] (Figure 2). In addition, in bladder cancer, progranulin interacts with the F-actin-binding protein drebrin [79]. In this tumor model, drebrin mediates progranulin-dependent cell migration and invasion by modulating F-actin remodeling [79]. Recently, we have demonstrated that in mesothelioma, progranulin regulates FAK phosphorylation, thereby modulating focal adhesion (FA) turnover, particularly FA disassembly, which is a critical step in cell motility [65].

#### 4.1.2. Progranulin and the Maintenance of CSC

Progranulin has been implicated in the maintenance of CSC, a subpopulation of tumor cells with stemness-like properties and tumor-initiating ability, often determining tumor recurrence [80,81]. Cheung et al. described progranulin as an oncofetal protein detected in fetal liver and hepatic cancer cell subpopulations expressing stemness markers, such as Nanog, Oct4 and Sox2, and showing an increased capacity to form tumors in vivo and induce resistance to chemotherapy [82]. In glioblastoma, progranulin sustained the expression of stemness genes, including *CD133*, *CD44* and *ABG2* [83]. In addition, progranulin depletion reduced self-renewal and multilineage differentiation capacity of R1S1 glioblastoma cells, contributing to temozolomide resistance [83]. In breast cancer, progranulin promoted proliferation of CSC and caused their dedifferentiation in a sortilin-dependent manner, suggesting a critical role for progranulin and sortilin in the maintenance of breast CSC [84,85].

### 4.2. Progranulin and the Tumor Microenvironment

#### 4.2.1. Progranulin in Tumor Angiogenesis and Lymphangiogenesis

Progranulin has an important role in physiological angiogenesis. Progranulin is expressed at low levels in quiescent endothelial cells, but progranulin expression is upregulated following endothelial cell activation during wound healing, tissue repair and physiological angiogenesis in the developing placenta [3,4]. Progranulin action in angiogenesis has been also demonstrated using transgenic mice. Indeed, progranulin overexpression in endothelial cells caused high rates of perinatal mortality because of expanded vessels size and progressive disruption of vascular integrity [6]. In many tumor models, progranulin has been detected in tumor-associated vasculature [51,86,87,88]. In colorectal cancer, progranulin promotes VEGF expression in a TNFR2/AKT/MAPK-dependent manner [89] and a similar action has been suggested in breast cancer cells, as well [90]. In agreement with the role of progranulin in promoting VEGF expression, progranulin levels positively correlate with VEGF expression and microvessel density in several tumor models, including breast carcinoma [90,91], esophageal squamous cell carcinoma [87] and colorectal cancer [89]. Notably, it has been suggested that progranulin might also promote angiogenesis in a VEGF-independent manner in mesothelioma [92]. In addition, progranulin interacts with the growth factor midkine (MK), a heparin-binding growth factor, and, in association with it, promotes HUVEC cells proliferation, migration and tubulogenesis [93]. Interestingly, it has been suggested that, in esophageal cancer, progranulin can also sustain lymphangiogenesis by favoring the expression of VEGF-C [94].

#### 4.2.2. Progranulin and Tumor Immune Evasion

Tumors develop multiple mechanisms to escape the host’s immune surveillance [95]. Growing evidence suggests that progranulin contributes to tumor immune evasion, not only by inhibiting immune cells but also by rendering tumor cells less immunogenic. Indeed, progranulin inhibits T lymphocytes proliferation and induces the generation of regulatory T lymphocytes (Treg) [96].

In hepatocellular carcinoma, progranulin rendered tumor cells resistant to natural killer (NK) cytotoxicity by promoting the downregulation of MHC class I chain-related molecule A (MICA) and upregulation of human leukocyte antigen E (HLA-E), the ligands of NK activator receptor NK group 2 member D (NKG2D) and NK inhibitory receptor CD94/NKG2A, respectively [97]. In agreement, progranulin inhibition restored NK cell activity [98].

In metastatic pancreatic cancer, macrophage-derived progranulin promoted CD8+ exclusion, contributing to tumor resistance to immune checkpoint inhibitors [99]. In the murine melanoma tumor model B16, progranulin promoted tumor growth by reducing recruitment of NK cells to the tumor microenvironment [100].

Notably, in breast cancer, progranulin promoted the expression of PD-L1 on tumor-associated macrophages (TAM) and favored their M2 polarization, leading to lymphocytes CD8+ exclusion [101]. In another study, exosomes derived from *GRN*^−/−^ TAM inhibited breast cancer cell migration and invasion [102]. Finally, in pancreatic ductal carcinoma, high progranulin levels are associated with reduced MCHI expression and a lack of CD8+T lymphocyte infiltration [103].

#### 4.2.3. Progranulin and Stromal Fibroblasts/Myofibroblast

The first evidence supporting progranulin action in modulating tumor stromal fibroblast function was reported by Elkabets et al. in 2011 [104]. The authors observed that MDA-MB-231 breast cancer cells subcutaneously implanted on one flank in mice promoted the expression of progranulin in Sca−/cKit−/CD45+ bone marrow-derived cells. The activated and progranulin-expressing Sca−/cKit−/CD45+ bone marrow-derived cells were then recruited to the site of the indolent tumor HMLER-HR, which was injected on the other flank, where they released progranulin, thereby stimulating expression and production of chemokines, cytokines, growth factors and matrix remodeling proteases by stromal fibroblasts and myofibroblasts, favoring growth and progression of these indolent tumors [104]. In a murine model of pancreatic ductal adenocarcinoma, Nielsen et al. demonstrated that metastasis-associated macrophages (MAMs) activated resident hepatic stellate cells into myofibroblasts by secreting progranulin, in turn creating a fibrotic TME suitable for metastatic tumor growth [105]. Interestingly, the authors also observed high expression levels of progranulin in hepatic MAMs and circulating monocytes derived from pancreatic ductal adenocarcinoma patients [105]. Finally, in colorectal cancer, tumor cell-derived progranulin has a role in promoting the conversion of fibroblasts into CAFs [106].

### 4.3. Diagnostic, Prognostic and Predictive Roles of the Progranulin Axis in Cancer

Progranulin is upregulated in many tumors, as compared to normal tissues, suggesting that progranulin can serve as a biomarker for several cancer types, including breast, prostate, ovarian, colon and bladder cancers, non-small cell lung carcinoma and brain tumors.

In breast cancer, progranulin has been proposed as a diagnostic, predictive and prognostic marker, as progranulin levels correlated with tumor angiogenesis, tumor size and the presence of metastasis in lymph nodes [107,108,109,110,111,112,113]. In addition, in patients with estrogen receptor-positive invasive ductal carcinoma, high progranulin levels in breast tumor tissue sections inversely correlated with disease-free tissue and overall survival rates and were predictive of recurrence risk and increased mortality [109]. Progranulin serum levels were higher in breast cancer patients when compared to healthy individuals and were predictive of recurrence in hormone-receptor-positive breast cancer patients treated with tamoxifen [114]. In metastatic breast cancer patients, progranulin serum levels were associated with disease progression and response to therapy [112]. Notably, Berger et al. reported that the co-expression of progranulin and sortilin identified a highly malignant subgroup of breast cancers [115].

Progranulin expression is higher in prostate tumors than in normal prostate tissue [116,117]. In prostate cancer patients, progranulin serum levels change with age and Gleason score, with lower progranulin serum levels being associated with better overall survival [118]. In addition, progranulin serum levels in combination with miR-486 levels might work as biomarkers predictive for therapy decisions in elderly prostate cancer patients [119]. Furthermore, progranulin expression in prostate cancer tissues is an independent prognostic factor for overall, disease-specific, and relapse-free survival in prostate cancer patients [120].

Similarly, ovarian epithelial cancers (EOC) showed progranulin upregulation as compared to normal ovarian tissues and a negative correlation between progranulin mRNA levels and poor overall survival in ovarian tumors [121]. Progranulin expression was demonstrated in both primary and metastatic EOC, as well as tumor stromal cells, and the presence of progranulin-positive stromal cells in untreated primary tumors was associated with reduced overall survival [86]. In addition, progranulin serum levels can have prognostic value for ovarian cancer patients [122], particularly in patients with advanced stages of EOC [123].

Colorectal cancer (CRC) tissues showed increased levels of progranulin as compared to normal colorectal tissues, and progranulin levels positively correlated with Ki67 and VEGF-A expression [89]. Furthermore, high progranulin levels were associated with poor recurrence-free survival in a retrospective analysis of CRC patients who underwent curative resection [124].

Progranulin is detectable in urine [125] and its levels are proposed as both diagnostic and prognostic markers for bladder cancer [126,127]. Recent data have indicated that progranulin levels in tumor cells and tumor-infiltrating immune cells likely work as prognostic markers in muscle-invasive urothelial bladder cancer, where high progranulin levels in tumor cells are considered a negative prognostic marker, while high progranulin levels in tumor-infiltrating immune cells are associated with better prognosis [128]. Interestingly, immunohistochemical analysis of progranulin and EphA2 expression showed progranulin and EphA2 upregulation in urothelial carcinoma tissues [125,129]. In addition, the expression of drebrin, a mediator of progranulin action in bladder cancer, is significantly higher in high grade versus low grade urothelial carcinoma tissues [79].

Progranulin expression is not detected in normal lung tissues or in small cell lung carcinoma, but it is expressed in lung adenocarcinoma, squamous cell carcinoma and non-small cell lung carcinoma (NSCLC) [110,130]. In NSCLC patients, progranulin tissue and serum levels are prognostic factors for recurrence [110,130], and high progranulin levels in bronchoalveolar lavage fluids of NSCLC patients were associated with shorter overall survival [131].

Progranulin levels were upregulated in astrocytoma and positively correlated with pathological grade [88]. In addition, a prognostic value was demonstrated for progranulin levels in glioblastoma patients [88]. Interestingly, progranulin levels increase in cerebrospinal fluids of patients presenting with lymphoma or carcinoma brain metastasis [132].

Finally, the potential use of progranulin as a prognostic marker is also currently under investigation in other tumors, such as oral squamous cell carcinomas [133], advanced biliary tract carcinoma [134], gastrointestinal tumors [135] and papillary thyroid cancer [136].

### 4.4. Progranulin Role in Tumor Resistance to Anticancer Therapies

Progranulin contributes to therapy resistance in many cancer types. However, the precise molecular mechanisms by which progranulin exerts this action are not completely understood.

The first report suggesting a role for progranulin in conferring resistance to chemotherapy was in breast cancer, as Tangkeangsirisin et al. observed that progranulin counteracted tamoxifen-induced apoptosis in breast cancer cells by inhibiting bcl-2 downregulation and preventing poly (ADP-ribose) polymerase cleavage [137]. It was later reported that, in Her-2 overexpressing breast cancer cells, progranulin conferred resistance to trastuzumab by promoting ErbB2/Her-2 phosphorylation [107]. In another study, the authors demonstrated that progranulin can also confer resistance to the aromatase inhibitor letrozole in breast cancer cells [138].

Several reports indicate that progranulin promotes resistance to platinum-based chemotherapy agents in various cancer types, including ovarian [139], colorectal [140], hepatocellular [141] and bladder cancer [142]. In hepatocellular carcinoma, a role for progranulin in promoting resistance to doxorubicin has also been demonstrated [141]. Progranulin-dependent expression of adenosine triphosphate–dependent binding cassette (ABC)B5 drug transporter is likely the potential molecular mechanism by which progranulin promotes tumor cell resistance to platinum-based and doxorubicin drugs [143].

In glioblastoma, progranulin promoted resistance to temozolomide by enhancing the expression of DNA repair and stemness genes [83].

In addition to chemotherapy, progranulin also contributes to radiation-therapy resistance, as reported in prostate cancer cells [144]. Finally, progranulin can contribute to tumor immune escape, thereby conferring resistance to immune checkpoint inhibitors [99].

### 4.5. Progranulin as a Therapeutic Target in Cancer

Progranulin’s pro-tumorigenic role makes it an attractive target for cancer therapy [16,60]. Many studies have demonstrated the efficacy of progranulin-inhibition in reducing in vitro tumor cell proliferation, migration and invasion, as well as in vivo tumor formation in multiple tumor models, as reviewed by Arechavaleta-Velasco et al. [74]. Current research is mostly focusing on the development of monoclonal neutralizing antibodies specific for progranulin. Notably, in February 2022, the first in-human phase 1 study of the anti-progranulin antibody AG01 [76] was started in patients with advanced solid tumors, particularly triple negative breast cancer, hormone-resistant breast cancer, NSCLC and mesothelioma patients (ClinicalTrials.gov Identifier: NCT05627960).

## 5. Progranulin Signaling in Cancer

Progranulin oncogenic signaling is highly dependent on AKT and/or MAPK pathways, which are the signaling cascades typically activated by growth factor receptors. Indeed, progranulin evokes the activation of AKT and MAPK signaling in many tumor models, including colorectal [89], bladder [59,78,79,129,142,145], breast [76], ovarian [121], prostate [117,146], cervical [147,148] and gastric cancers [149], hepatocellular carcinoma [150,151], NSCLC [152], esophageal cell squamous carcinoma [153], cholangiocarcinoma [75,154] and mesothelioma [65]. AKT and MAPK activation are key events in progranulin oncogenic action, since these two signaling pathways are essential for cell proliferation and survival, migration and invasion [155] (Figure 2).

**Figure 3:**
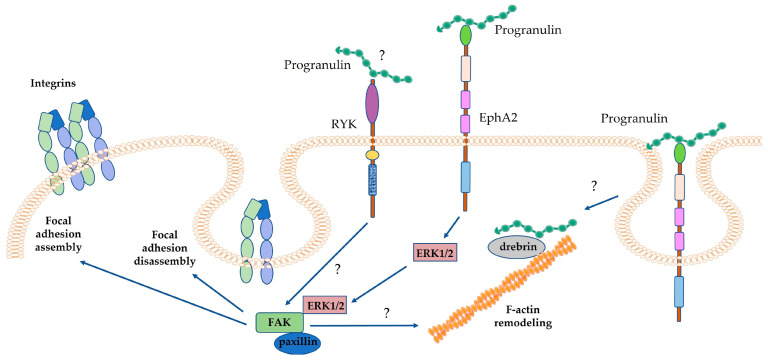
Progranulin modulates FAK activity. In bladder cancer, progranulin-dependent activation of ERK1/2 promotes the formation of a complex containing FAK, paxillin and ERK1/2, thereby promoting cell motility. In addition, in bladder cancer, progranulin interacts with the F-actin-binding protein drebrin, promoting F-actin remodeling. However, the mechanism by which progranulin interacts with drebrin is still unknown and could be dependent on receptor-mediated progranulin internalization. In mesothelioma cells, progranulin modulates the phosphorylation of FAK, affecting the dynamics of focal adhesion assembly/disassembly and F-actin remodeling. RYK action in progranulin-dependent modulation of FAK in mesothelioma is still not well defined.

Progranulin-mediated regulation of cell motility also relies on FAK activity (Figure 3). Indeed, in adrenal carcinoma cells, progranulin promotes FAK tyrosine-phosphorylation [4]. Furthermore, in bladder cancer, progranulin-dependent activation of MAPK favors the formation of a complex containing paxillin and FAK, thereby promoting cell migration and invasion [78]. Recently, we have demonstrated that in mesothelioma cells, progranulin modulates the phosphorylation of FAK at Y397, affecting focal adhesion kinetics and, more specifically, the process of FA assembly/disassembly [65]. Progranulin-dependent regulation of FA turnover is likely the mechanism by which progranulin influences mesothelioma cell motility [65]. Since FAK is a key mediator of integrin signaling, these data might also suggest a potential role for progranulin in modulating integrin function. There are some indications that this might be the case, as in fact it has been demonstrated that progranulin promoted prostate cancer cells’ adhesion to bone marrow endothelial cells (BMEC) in an NF-kB and integrin-α4-dependent manner [156]. In addition, integrin-α3 was among the potential progranulin membrane binding proteins recently identified in NSC-34 cells by Chitramuthu et al. [48].

In addition to AKT, ERK1/2 and FAK, progranulin can also sustain the activity of signal transducer and transcription activator3 (STAT3) [157]. Indeed, in colorectal cancer cells, progranulin physically interacted with STAT3, evoking its phosphorylation and pro-oncogenic downstream signaling [157].

Although progranulin-dependent activation of AKT and MAPK and, to a lesser extent, FAK and STAT3 has been extensively demonstrated, how progranulin leads to their activation is not fully defined and evidence suggests context-dependent mechanisms (Figure 2). In colorectal cancer cells and in human vascular endothelial cells, TNFR2 is required for progranulin-dependent stimulation of the AKT pathway [89] (Figure 2). On the other hand, in breast cancer, progranulin action is mediated by sortilin, as, in fact, progranulin promoted breast cancer CSC’ expansion in a sortilin-dependent manner [84]. In agreement with a role for sortilin in supporting progranulin oncogenic action, sortilin inhibition counteracted progranulin-dependent breast cancer progression and CSC expansion [84,85]. Furthermore, co-expression of progranulin and sortilin might work as a biomarker, which identifies a highly malignant subgroup of breast cancers [115]. By contrast, in prostate cancer cells, sortilin acts as a negative modulator of progranulin activity, as its overexpression reduced progranulin levels by promoting clathrin-dependent progranulin internalization and degradation, leading to a reduction in AKT activation, cell proliferation, migration, invasion and anchorage-independent growth [146,158,159] (Figure 2). Significantly, we later demonstrated that progranulin downregulated sortilin protein levels independently of transcription by mediating sortilin ubiquitination, internalization via clathrin-dependent endocytosis and trafficking into early endosomes for lysosomal degradation. These results suggest a fine-tuned regulatory feedback mechanism, whereby sortilin downregulation ensures sustained progranulin-mediated oncogenic action in prostate cancer [159]. However, whether this regulatory mechanism is conserved in other tumor models requires further investigation. Interestingly, in bladder cancer, the F-actin-binding protein drebrin interacts with progranulin and is involved in mediating progranulin-dependent activation of the AKT and MAPK pathways [79].

An important step forward in deciphering progranulin oncogenic mechanisms of action was the identification of EphA2 as the functional progranulin receptor in bladder cancer [59]. EphA2 is a member of the Eph family of RTKs and its role in cancer is controversial. EphA2 activation by its canonical ligand, ephrin-A1, evokes EphA2 canonical signaling inhibiting cancer cell migration and invasion [160]. Conversely, ephrin-A1-independent and AKT- or RSK-dependent phosphorylation of EphA2 at Ser 897 determines EphA2 pro-oncogenic activity [161,162]. In bladder cancer, progranulin binds to and triggers EphA2 tyrosine-phosphorylation, with consequent activation of the AKT and MAPK signaling pathways, which in turn promote EphA2 phosphorylation at Ser 897 [59,129] (Figure 2). In this tumor model, the progranulin/EphA2 axis drives tumor cell migration, invasion, anchorage-independent growth, in vivo tumor formation and cisplatin-resistance [129].

Recently, we have demonstrated that in mesothelioma cells, EphA2 is not the major progranulin signaling receptor and progranulin action is instead mediated by EGFR and RYK, a co-receptor of the Wnt pathway [65] (Figure 2). Notably, in this tumor model, progranulin sustains AKT and MAPK activation and the phosphorylation of EphA2 at Ser 897, as in bladder cancer cells. However, the contribution of EphA2 activation is not clearly defined in mesothelioma cells, where we identified by RTK arrays that progranulin promoted tyrosine-phosphorylation of EGFR and RYK. Significantly, in this experimental approach, we did not detect any Tyr-phosphorylation of EphA2 [65]. Progranulin-dependent EGFR activation was not totally surprising, as it has been observed in other models, including bladder cancer [59], breast cancer [84] and mammary epithelial cells [48]. However, we do not know whether progranulin modulates EGFR activity directly, by physically interacting with the receptor, or in an indirect manner. The modulation of RYK activity by progranulin is of particular interest. RYK is a Wnt-binding RTK with a role as a co-receptor for both canonical (β-catenin-dependent) and non-canonical (β-catenin-independent) Wnt signaling pathways [163]. Interestingly, RYK does not likely have kinase activity, suggesting that RYK action depends on functional interactions with other receptors. Indeed, it has been demonstrated that RYK forms complexes with Frizzled (FDZ) receptors, but also with other RTKs, such as Eph receptors [163]. There are data suggesting that some Eph receptors can mediate RYK phosphorylation [164,165], but the functional relevance of RYK interaction with other RTKs is still unknown. It is tempting to hypothesize that, in mesothelioma, EGFR could be involved in progranulin-stimulated RYK tyrosine-phosphorylation and that progranulin signaling might depend on EGFR and RYK physical and functional interactions. In addition, because EGFR modulates EphA2 phosphorylation at Ser897 in mesothelioma cells [65], we can also hypothesize that EGFR could promote RYK phosphorylation indirectly by modulating EphA2 activity (Figure 2). The potential role played by RYK in cancer is, at the moment, not well defined, but there are a few studies demonstrating increased RYK expression in some tumor models, such as glioblastoma [166], acute lymphoblastic leukemia and acute myeloid leukemia [167] and others [163]. In addition, a role for RYK in mediating cell migration and anchorage-independent growth in cancer cells has been suggested [166]. Thus, it would be interesting to investigate whether progranulin oncogenic action is mediated, at least in part, by RYK and the Wnt pathway. Notably, previous reports suggested that progranulin might modulate the Wnt pathway, as in fact there is a correlation between progranulin haploinsufficiency and dysregulation of Wnt signaling [168,169,170,171,172]. Interestingly, Rosen et al. demonstrated that FTD caused by *GRN* haploinsufficiency is partially mediated by changes in Wnt signaling [168]. Notably, Wnt pathway dysregulation, characterized by the upregulation of genes belonging to Wnt canonical signaling and downregulation of negative regulators of Wnt signaling, is an early event in *GRN* haploinsufficient FTD and precedes the onset of the neurodegenerative process [168,172]. However, how progranulin regulates the Wnt pathway is not yet defined. Most of the studies establishing a connection between *GRN* haploinsufficiency and Wnt dysregulation focused on neuronal cells derived from animal models or patients affected by frontotemporal dementia [168,169,170], but there are also studies investigating other pathological conditions associated with a reduction in progranulin levels, such as intervertebral disc degeneration [171]. It would be interesting to investigate whether RYK might have a role in this context, and whether progranulin might either interfere or potentiate Wnt signaling pathways in cancer by functionally interacting with RYK.

Finally, progranulin can also activate additional receptors, including other members of the Eph family of RTKs [48,59], ErBB2 and RET [48]. Whether these receptors might contribute to progranulin oncogenic action remains unexplored. Overall, these data suggest a complex modulation of progranulin oncogenic signaling, which could depend on progranulin-mediated crosstalks between multiple RTKs depending on cellular context.

## 6. Conclusions and Future Perspectives

Growing evidence supports a critical role for progranulin in cancer, both as a pro-oncogenic molecule and a theragnostic biomarker, thereby making it an attractive target for cancer therapy. Recent studies suggest that progranulin mechanisms of action are highly context dependent and involve the activation of multiple RTKs and downstream signaling pathways. This aspect of progranulin activity suggests that progranulin-based therapeutic approaches might have to be tailored to specific tumor contexts and that multimodal approaches might be required to target the multiple signaling pathways that are activated by progranulin.

## Figures and Tables

**Table 1:** Progranulin action in cancer. For references, see [15,74] and references throughout the text.

**Progranulin Autocrine Function on Tumor Cells**	
Cell proliferation	Colorectal cancer, lung carcinoma, cervical cancer, prostate carcinoma, adrenal carcinoma, laryngeal carcinoma, breast carcinoma.
Cell migration and invasion	Breast cancer, colorectal cancer, bladder cancer, prostate carcinoma, adrenal carcinoma, hepatocellular carcinoma, ovarian carcinoma, mesothelioma.
CSC maintenance	Hepatocellular carcinoma, breast carcinoma, glioblastoma.
**Progranulin Modulation of the Tumor Microenvironment**	
Tumor angiogenesis and lymphangiogenesis	Colorectal cancer, breast cancer, mesothelioma, esophageal squamous cell carcinoma.
Tumor immune evasion	Hepatocellular carcinoma, metastatic pancreatic cancer, pancreatic ductal carcinoma, breast carcinoma.
Stimulation of fibroblasts and myofibroblasts function	Breast carcinoma, pancreatic ductal adenocarcinoma, colorectal carcinoma.
**Progranulin Axis as a Biomarker in Cancer**	
Diagnostic and/or prognostic and/or predictive marker	Breast carcinoma, prostate carcinoma, ovarian epithelial cancers, colorectal carcinoma, bladder cancer, non-small cell lung carcinoma, astrocytoma, glioblastoma, oral squamous cell carcinomas, biliary tract carcinoma, gastrointestinal tumors, papillary thyroid cancer.
**Progranulin and Resistance to Anticancer Therapies**	
Chemotherapy	Breast carcinoma, ovarian, colorectal, and hepatocellular carcinomas, glioblastoma, bladder cancer.
Radiation therapy	Prostate cancer.
**Progranulin as a Therapeutic Target in Cancer**	
Progranulin inhibition via genetic depletion or neutralizing antibodies	Breast carcinoma, ovarian cancer, hepatocellular carcinoma, bladder cancer.
